# Clinicopathological findings of FeLV- positive cats at a secondary referral center in Florida, USA (2008–2019)

**DOI:** 10.1371/journal.pone.0266621

**Published:** 2022-04-07

**Authors:** Amelie Pare, Alexandre Ellis, Tristan Juette

**Affiliations:** 1 Department of Internal Medicine, Western Veterinary Specialist & Emergency Centre–VCA, Calgary, Alberta, Canada; 2 Shelter Outreach Consultation Services, Calgary, Alberta, Canada; 3 Faculty of Veterinary Medicine, University of Montreal, St-Hyacinthe, Quebec, Canada; University of Lincoln, UNITED KINGDOM

## Abstract

**Objectives:**

The aim of this study was to describe the seroprevalence, presenting complaint, clinicopathological changes, co-morbidities and outcomes of feline leukemia virus positive cats presented to a specialty referral center in Florida, USA.

**Methods:**

In this retrospective study, medical records of 8050 cats presented to a private referral center from August 2008 to September 2019 were reviewed. Inclusion criteria required was a positive result for feline leukemia virus by point-of-care antigen testing or immunofluorescence assay.

**Results:**

Forty-one cases met the inclusion criteria. Of 2002 cats that were tested, 41 cats (2%) met the inclusion criteria. One cat had a negative point of care antigen test result and positive bone marrow IFA result. The mean age at diagnosis was 9 years. The main reasons for presentation were abnormal complete blood cell count results (35%), followed by pleural effusion (18%), and anorexia (15%). The most common laboratory abnormalities included anaemia (71%), of which 74% had a nonregenerative anemia, thrombocytopenia (52%), elevated aspartate aminotransferase (50%), hyperbilirubinemia (35%), and hypokalemia (35%). Seven percent of cats (3/41) were also positive for feline immunodeficiency virus. The most common diagnoses were neoplasia (76%) and bone marrow disorders (12%). Cats with neoplasia were significantly younger. Survival to discharge was 88%.

**Conclusion and relevance:**

Results of this study show that feline leukemia virus is uncommon in secondary referral center, even if this represents a population of unhealthy cats. The most common associated diagnosis was neoplasia, which was more likely to be seen in younger cats (< 4 years of age). The mean age of cats positive for feline leukemia virus was also older than previously published data. These findings support the current guidelines which indicate that cats presented with clinical illness should be tested for FeLV at the time of presentation.

## Introduction

Feline leukemia virus (FeLV) is a *Gammaretrovirus* that has been associated with neoplasia, bone marrow disorders, immune-mediated diseases and immunodeficiency in cats [1]. Historically, it has been seen mostly in sick, young, feral cats and was thought to be the most common cause of neoplasia in cats [2–5]. Studies have shown a significant decrease in the importance of FeLV-associated lymphoma since vaccination has been made widely available and has been recommended for cats based on their lifestyle [3,6,7].

Feline leukemia virus is transmitted through oronasal secretions, grooming, bite wounds or by vertical transmission from a queen to her kittens [3]. Infection can be categorized as an abortive infection (elimination of the virus due to an appropriate immune response), a progressive infection (characterized by a persistent viremia) or a regressive infection (the viremia is eliminated, but proviral DNA remains in the cat’s genome) [3]. The first of several inactivated FeLV vaccines was introduced in the 1980s, and current vaccination guidelines advocate for FeLV vaccination in all cats younger than 1 year of age as well as cats that are considered at higher risk due to their lifestyle or other factors [6,8,9]. This includes cats who roam outdoors, households where new cats are frequently introduced or in cats living with known FeLV infected cats [3]. In addition to vaccination, the identification and segregation of infected cats has been an important factor in limiting the spread of disease. The most common diagnostic methods involve detection of FeLV p27 capsid antigen with point-of-care (POC) testing with either ELISA or immunochromatography assays. Other common diagnostic assays include IFA that detects intracellular FeLV p27 antigen within infected neutrophils and platelets, and PCR that can detect FeLV proviral DNA and plasma viral RNA. Testing of the bone marrow or blood via PCR can increase the probability of detection of the virus in cases where there is no active viremia [3,10].

Previous retrospective studies showed that overall prevalence of FeLV is between 3.1% and 3.4% for healthy cats, but up to 30% in unhealthy cats in North America [11–13]. These numbers have been decreasing since the virus was first identified in Scotland in 1964 [3,14–16]. Based on this trend, it could be inferred that other clinical aspects of the disease might have changed as well during the past decades. To the authors’ knowledge, there is no study reporting prevalence or clinicopathological findings of cats presented to a referral center within the past 10 years. The goal of this study was to describe the prevalence, presenting complaints, final diagnosis, clinicopathological changes, co-morbidities, and outcomes of clinically ill FeLV-positive cats presented to a specialty referral center in Florida through the past decade (2008–2019).

## Materials and methods

This study describes data retrieved from medical records from owned animals that received standard of care as part of their consultation. Therefore, owner informed consent and ethical approval from a committee was not specifically obtained for this manuscript due to its retrospective nature.

A retrospective analysis of medical records of all cats presented to a referral hospital in Florida, USA, Affiliated Veterinary Specialist, between January 2008 and September 2019 was performed. The data was reviewed, and inclusion criteria required a positive result for FeLV via a POC test on blood, either with the referring veterinarian or at the referral center, or a bone marrow IFA. Retrovirus testing done at the referral center was performed with POC ELISA for FeLV p27 antigen and feline immunodeficiency virus (FIV) antibodies to p15 and p24 (SNAP® FIV/FeLV Combo Test, IDEXX Laboratories, Inc., Westbrook, ME, USA). Reported sensitivity by the manufacturer are 98.6% for FeLV and 93.5% for FIV in unvaccinated cats [14,17]. Specificity Immunofluorescence bone marrow samples were sent to a third-party laboratory (IDEXX Laboratories, Inc., Westbrook, ME, USA).

Data extracted from the medical records included signalment (age, sex, neuter status and breed), presenting complaint, final diagnosis (when available), and survival to discharge. Results from complete blood count (CBC) and serum chemistry at the time of diagnosis were recorded when available. All blood samples were sent to a third-party laboratory (IDEXX Laboratories, Inc., Westbrook, ME, USA) for analysis and all CBCs were reviewed manually for possible platelet clumping at the laboratory.

### Statistical analysis

Variables were reported as frequencies and percentages. Data regarding the CBC and serum chemistry values were reported as mean values. The range (minimum-maximum) and 95% confidence intervals (CI) for those were also calculated.

The normality of the data regarding CBC, serum chemistry, age and final diagnosis for all dependent variables was determined using a Shapiro-Wilk test. For normal variables, Levene’s test was used to determine the homoscedasticity associated with categorical explanatory variables. To evaluate the correlation between age and the presence of neoplasia, the data was not normally distributed, and a Spearman’s rank correlation was used. For other tests, the explanatory variable was always the presence of neoplasia, which is a categorical variable, with two groups (presence or absence). In accordance with results of the Shapiro-Wilk and the Levene tests, parametric t-tests for some dependent variables (hematocrit, potassium, chloride, albumin, calcium, and phosphorus) were performed. For all other dependent variables with a non-parametric distribution, the Mann-Whitney test was used. For all tests, the significance threshold was set to 0.05. Statistical analyses were run with the software R version 4.0.3 (R Core Team. R: A language and environment for statistical computing. R Foundation for Statistical Computing, Vienna, Austria).

## Results

Records from 8050 cats presented to the practice were reviewed. Of those, 2002 had evidence of testing for FeLV at presentation at the referral center or with their referring veterinarians prior to presentation. Forty-one met the inclusion criteria, which resulted in a prevalence of 2% (41/2002) in the population of tested cats. Diagnostic work-up differed between the 41 cases, and one cat had incomplete available medical records; relevant information from each case was included when applicable.

### Signalment and presenting complaint

Mean age of cats was 9 years (range 1 to 21 years). There were 19 males and 22 females; all were neutered. The most common breed was domestic cats (39/41); the others were Savannah (1/41) and Siamese (1/41). The presenting complaints were available in 40 cats and are described in Table 1. The most common were anemia (11/41; 27%), pleural effusion (7/41; 17%) and anorexia (6/41; 15%).

**Table 1:** Presenting complaints of FeLV-positive cats.

Presenting Complaint	N (%)
**Anemia**	11/41 (26.8)
**Pleural effusion**	7/41 (17.1)
**Anorexia**	6/41 (14.6)
**Pancytopenia**	3/41 (7.3)
**Respiratory distress**	2/41 (4.9)
**Lethargy**	2/41 (4.9)
**Urinary incontinence**	1/41 (2.4)
**Seizures**	1/41 (2.4)
**Abdominal mass**	1/41 (2.4)
**Paraparesis**	1/41 (2.4)
**Neck pain**	1/41 (2.4)
**Draining cutaneous mass**	1/41 (2.4)
**Chronic nasal discharge**	1/41 (2.4)
**Fever**	1/41 (2.4)
**Unregulated diabetes**	1/41 (2.4)
**Investigation of FeLV diagnosis at referring veterinarian**	1/41 (2.4)

### Diagnostics

Initial diagnosis of FeLV was made via a POC ELISA in 37/41 cats (90%) and via IFA of a bone marrow sample in 4/41 cats (10%). Of the cats positive on IFA, two presented for pancytopenia and two presented for anemia. Only one of the four had evidence of a negative POC ELISA test result beforehand. The other three cats did not have evidence of POC testing at the referral practice. None of the cats were tested via PCR.

Hematologic (n = 28) and biochemical (n = 20) data were evaluated and presented in Table 2. Anemia was defined as a hematocrit below 29% and was identified in 20/28 (71%) of cats. Of those, 19 had a reticulocyte count present; five were classified as regenerative, and 14 were classified as non-regenerative based on reticulocyte count less than 50 K/uL.

**Table 2:** Blood work findings of cats testing positive for FeLV.

Variable	RI	Median	Range
**HCT** (%)	29–45	22.6	6.7–49.4
**MCV** (fL)	36–59	52	16–89
**NRBC**	0–2	0	0–139
**Reticulocytes** (K/uL)	3–50	17	3–275
**WBC** (K/uL)	4.2–15.6	9	0.2–134.9
**Neutrophils** (/uL)	2620–15170	6924	27–94430
**Bands** (/uL)	0–300	0	0–12141
**Eosinophils** (/uL)	90–2180	160	0–3708
**Basophils** (/uL)	0–100	0	0–40
**Lymphocytes** (/uL)	850–5850	20026	120–24282
**Monocytes** (/uL)	40–530	507	40–4047
**Platelets** (K/uL)	170–600	166	15–380
**AST** (U/L)	5–55	52	15–267
**Total bilirubin** (mg/dL)	0–0.4	0.2	0.1–8
**Potassium** (mmol/L)	3.9–5.3	4.1	2.4–5.5
**Albumin** g/dL)	2.3–3.9	3	1.9–4.1
**ALT** (U/L)	28–100	64	25–635
**Globulin** (g/dL)	3–5.6	3.6	1.6–7.6
**TT4** (ug/dL)	0.8–4.7	1.5	0.4–4.1
**Creatinine** (mg/dL)	0.8–2.3	1.2	0.9–2.3

RI: Reference interval, HCT: Hematocrit, MCV: Mean corpuscular value, NRBC: Nucleated red blood cells, WBC: White blood cells, AST: Aspartate aminotransferase, ALT: Alanine aminotransferase, TT4: Total thyroxine, CI: Confidence interval 95%.

The most prevalent serum biochemistry abnormalities included elevated aspartate aminotransferase (AST) in 10 cats (50%), hyperbilirubinemia in 7 cats (35%), hypokalemia in 7 cats (35%), elevated alanine aminotransferase (ALT) in 6 cats (30%) and hypoalbuminemia in 5 cats (25%).

### Comorbidities

Only 8/41 (20%) cats had comorbidities present including 3/41 (7%) concurrently positive for FIV. Other comorbidities included diaphragmatic hernia (n = 1), skin allergies (n = 1), keratitis (n = 1), chronic kidney disease in addition to hyperthyroidism (n = 1) and hypertrophic cardiomyopathy (n = 1).

### Final diagnosis

**Fig 1:**
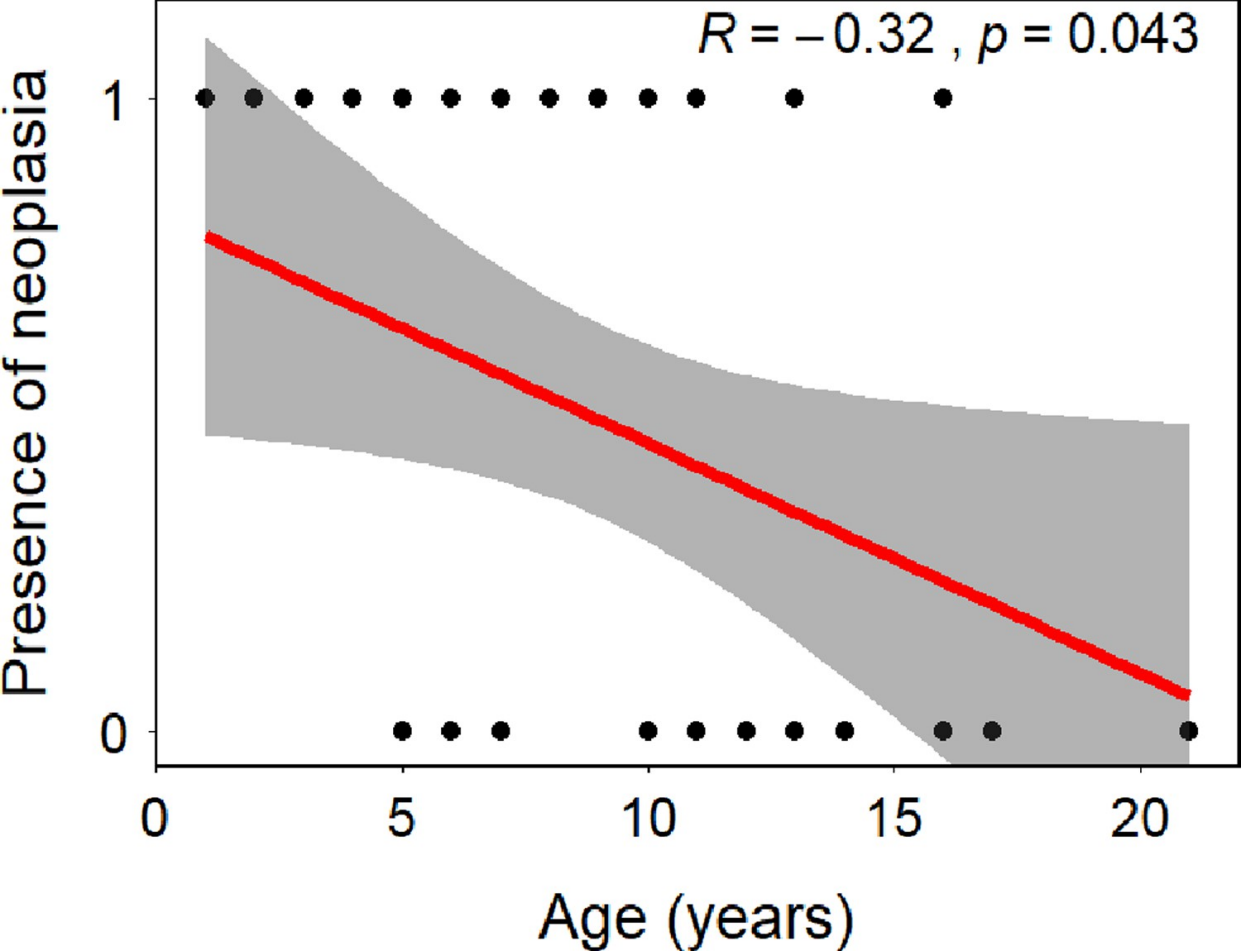
Correlation (+ CI) between the age of the individuals and the presence of neoplasia. Statistics indicate Spearman’s rho and the p-value associated with the correlation.

Final diagnoses were available in 25 cats and described in Table 3. Nineteen (76%) were diagnosed with neoplasia via imaging and/or cytology. Of those, 13 had evidence of lymphoma (68%) based on cytology results: five had mediastinal lymphoma, 4 had multicentric lymphoma, 2 had renal lymphoma, 1 had a spinal lymphoma and 1 had myeloid lymphoma. Of the six cats with non-lymphoma neoplasia, two had acute myeloid leukemia based on bone marrow cytology, one had multiple myeloma, one had a sarcoma and two had masses that appeared grossly neoplastic via imaging but were not diagnosed by histopathology or cytology. The other most common diagnoses were non-neoplastic bone marrow disorders, which were present in 3 cats (12%). All cats younger than 4 years of age were diagnosed with neoplasia and there was a significant negative association (R = -0.32, p = 0.043) between age and the diagnosis of neoplasia (Fig 1). There was no significant association observed between CBC values and the presence or absence of neoplasia (Table 4).

**Table 3:** Final diagnosis in cats positive for FeLV.

Final Diagnosis	Nunmber (%)	Median age (years)	Range
**Neoplasia**	**19/25 (76)**	**7.5**	**1–13**
*Lymphoma*	13 (52)		
Mediastinal lymphoma	5 (20)		
Multicentric lymphoma	4 (16)		
Renal lymphoma	2 (8)		
Spinal lymphoma	1 (4)		
Myeloid lymphoma	1 (4)		
*Acute myeloid leukemia*	2 (8)		
*Multiple myeloma*	1 (4)		
*Ileo-colic mass*	1 (4)		
*Cervical sarcoma*	1 (4)		
*Mediastinal Mass*	1 (4)		
**Non-neoplastic bone marrow disorders**	**3/25 (12)**	**12**	**10–17**
*Dyserythropoiesis*	1 (4)		
*Erythroid hypoplasia*	1 (4)		
*Precursor targeted immune mediated anemia*	1 (4)		
**Other diagnosis**	**3/25 (12)**	**13**	**11–21**
Asthma	1 (4)		
Cholangitis and pancreatitis	1 (4)		
Cystic mass with marked mixed inflammation	1 (4)		

**Table 4:** Effect of the presence of neoplasia on CBC findings in FeLV positive cats compared to those without neoplasia.

Dependent variable	Value	df	p-value
HCT	0.719	25.641	0.479
MCV	63	/	0.116
NRBC	64.5	/	0.113
Reticulocytes	61	/	0.369
WBC	82.5	/	0.504
Neutrophils	71	/	0.235
Bands	71.5	/	0.054
Eosinophils	119	/	0.333
Basophils	120	/	0.183
Lymphocytes	95	/	0.927
Monocytes	81	/	0.467
Platelet	76	/	0.82

HCT: Hematocrit, MCV: Mean corpuscular value, NRBC: Nucleated red blood cells, WBC: White blood cells, df: Degrees of freedom.

The correlation between the presence of neoplasia and serum chemistry values are described in Table 5. Cats with neoplasia were more likely to have a significant decrease in globulins (mean 2.95 vs 4.33 g/dL, p = 0.004) and TT4 (mean 1.14 vs 1.94 ug/dL, p = 0.036).

**Table 5:** Effect of the presence of neoplasia on blood chemistry findings in FeLV positive cats compared to those without neoplasia.

Dependent variable	Value	df	p-value
Sodium	43	/	0.645
Potassium	0.328	16.428	0.747
Chloride	1.075	17.313	0.297
Albumin	-1.861	17.339	0.08
**Globulin**	**87.5**	**/**	**0.004**
Calcium	-0.348	17.939	0.732
CK	55	/	0.386
**TT4**	**62**	**/**	**0.036**
Creatinine	72	/	0.092
BUN	49	/	1
Phosphorus	-0.819	13.424	0.427
ALT	33.5	/	0.239
ALP	37.5	/	0.382
AST	29	/	0.128
Bilirubin	44	/	0.692

Bold lines indicate significant effects (p <0.05).CK: Creatine kinase, AST: Aspartate aminotransferase, ALT: Alanine aminotransferase, ALP: Alkaline phosphatase, TT4: Total thyroxine, df: Degree of freedom.

### Outcome

Status at discharge was available in all cats; 36/41(88%) survived to hospital discharge. All four euthanized cats had evidence of a mass or a diagnosis of neoplasia and were euthanized due a poor prognosis, poor quality of life and/or financial constraints of the owner. All four cats were determined to be FeLV-positive via POC testing. Further follow-up of discharged cats was not available given the retrospective nature of the study and palliative route chosen by most owners.

## Discussion

Several important new findings and differences from previously reported data were noted in this study, including the suspected prevalence, concurrent diagnosis, and age at presentation.

In this study, only 25% of cats presented to the referral center had evidence of current testing for retroviral infection at the time of presentation even though guidelines indicated testing for leukemia in any sick cat, regardless of their status before the onset of clinical signs [18]. This is concordant with previous findings, which showed a poor compliance of veterinarians with the recommendations to test for retroviral infections in newly adopted cats or cats with injuries [19]. Regressive infections, disease incubation during initial testing, and limitations in test sensitivity can all cause false-negative test results on POC and laboratory testing [3]. As such, serial testing or alternate diagnostic assays should be considered when there is a strong clinical suspicion of disease or a known recent exposure [3,19]. The findings in this article support that retroviral testing is indicated in sick feline patients regardless of their previous status, even if signs are not consistent with a classical presentation for FeLV.

The mean age at presentation was 9 years, which is more than twice the previously reported age in previous studies [4,20,21]. This could indicate that cats that are FeLV-positive tend to live longer than previously described. However, since younger cats were at higher risk of neoplasia in our study, this could also be a representation of survivorship bias in cats presented to a referral practice or could indicate that the virus affect different population of cats in different ways. Further research to determine if young cats are more likely to develop neoplasia secondary to FeLV than adult cats would be needed.

Even though causality cannot be confirmed, neoplasia was the most common diagnosis in this study’s population, which is in contrast to a 1991 study that reported coinfections and anemia as the two most common findings [22]. These earlier results are consistent with another retrospective study during the same time period which showed that only 23% of FeLV-positive cats positive died of tumor-related reasons, versus 76% in the present study [2].

The most common neoplasia noted in our population data was lymphoma. FeLV-associated lymphoma includes multicentric lymphoma, mediastinal lymphoma, neurolymphoma, and ocular lymphoma [7]. However, two cats in this study were diagnosed with renal lymphoma, which is typically a non-retrovirus associated neoplasia and could indicate an incidental diagnosis instead of a true causality. Intestinal lymphoma was not found in any cat in this study. Overall, 39% of cats with lymphoma had mediastinal involvement which is higher than previously reported (6%) [7]. Interestingly, mediastinal lymphoma was only seen in cats of 5 years of age or older, while it is reported the be more prevalent in younger cats in literature [23]. Furthermore, one cat of one year of age had a mediastinal mass for which further diagnostics were not pursued by the owners and could have been a presentation of mediastinal lymphoma.

All five cats younger than 4 years old were diagnosed with a neoplastic process and there was a significant negative correlation between age and the presence of neoplasia. There are many hypotheses to explain this relationship, including a causative relationship. Another explanation is that young cats with neoplasia are more likely to be tested for retroviruses due to the decreased incidence of neoplasia in that age group, skewing our population. This hypothesis supported by our observation of poor compliance with testing guidelines in all sick and injured cats.

The most common presenting complaints for cats testing positive for FeLV in this study were abnormal CBC results, pleural effusion, and anorexia. These presenting complaints were expected, since the two most common diagnoses were neoplasia and bone marrow suppression. To the authors’ knowledge, previous studies did not report presenting complaints and therefore those results cannot be compared.

The most common hematological change was anemia (71% of cats). Anemia in FeLV-positive cats is most commonly associated with bone marrow suppression from the virus affecting the hematopoietic stem cells as well as the surrounding stroma cells [1,4]. Other mechanisms suspected to result in anemia are inflammation from chronic disease, immune-mediated disease, secondary infections, nutritional deficiencies, or hematopoietic neoplasia. Six cats (21%) in the present study had an elevated MCV and this finding is suspected to arise from a defect in erythroid maturation. A previous study reported elevated MCV in up to 53% of cats with FeLV [24]. The second most common abnormal finding on the CBC was thrombocytopenia in 52% of cats. This abnormality in FeLV-positive cats is thought to originate from decreased production by the bone marrow or from leukemic infiltration.

Elevation of liver enzymes was the most common finding on serum chemistry. Interestingly, this study reports hyperbilirubinemia and elevated liver enzymes (ALT, AST) in more than 30% of cats. On results from necropsy of cats with FeLV in 1989, only 15% had signs of liver degeneration [2]. From 1997–2002, a retrospective study showed that changes in serum biochemistries of cats infected with FeLV did not significantly differ when compared to a control group [4]. Hypokalemia was noted in 35% of cats and is likely to be a result of malnutrition and gastrointestinal loss secondary to vomiting and diarrhea. In our study, we also found that cats with neoplasia had a significantly lower count of globulin and TT4 levels. However, neoplasia is usually associated with a higher globulin count [25]. As such, this finding is surprising and may be a combination of malabsorption and immunodeficiency more pronounced in this younger population. The lower TT4 might be secondary to sick-euthyroid syndrome or the younger median age of cats with neoplasia.

In the general population, consisting of healthy and unhealthy cats, 0.1–0.5% tested positive for both FIV and FeLV [4,11,12]. In one study, coinfection was reported in up to 1.3% of FeLV-positive cats [15]. The percentage of coinfection in this study was surprisingly much higher (7%). This might partially be due to the secondary referral nature of the practice, which more commonly sees severely affected animals as coinfection of retroviruses might predispose cats to more severe disease. It is important to note that FIV vaccination was available in the United States until 2017 and vaccination status of cats was not available in all cases. Point-of-care testing for FIV used in this study detects antibodies and cannot discriminate between vaccinated and infected cats [14]. However, the FIV vaccine was not considered a core vaccine, and, in the authors’ opinion, was a rarely administered vaccine when it was commercially available [26]. As such, there is little evidence to suggest that false-positive results skewed these results.

The limitations of this study are mostly owed to its retrospective aspect and low number of cases meeting the inclusion criteria. Furthermore, cats were recruited from a single practice in Florida for which local vaccination practices and owner demographics might alter results in different communities or different types of veterinary practices. Lifestyle habits, outcome and advanced diagnostics were not available for all cats, which limited the ability to make comparisons. In most cases, no additional testing (IFA, PCR) for FeLV status was performed and the type of infection (regressive vs progressive) could not be determined. This means that causality between final diagnosis and FeLV positive test cannot be established in our population due to the retrospective nature of the study and the aforementioned lack of additional testing.

## Conclusion

The present study reports the clinical presentation of cats with FeLV, in a specialty referral practice in Florida. Clinicians should strongly consider FeLV diagnostics in any unhealthy cat, especially presenting with a nonregenerative anaemia, thrombocytopenia, elevated aspartate aminotransferase and lymphoma, and particularly in young cats presenting with neoplasia of haematopoietic origin. FeLV infection status should be confirmed in cats testing positive for FeLV p27 antigen using FeLV PCR testing or referral laboratory microtiter antigen test or IFA on appropriately collected specimens. Prospective studies using quantitative PCR could be useful to determine if clinical presentation differs based on virus load.

## Supporting information

S1 Fig Data available for all cats. MN: Male neutered, FS: Female spayed, Y:Yes, N:No, n/a: No applicable.
(XLSX)

S2 Fig CBC results. HCT: Hematocrit, MCV: Mean corpuscular value, NRBC: Nucleated red blood cells, WBC: White blood cells.
(XLSX)

S3 Fig Chemistry results. CK: Creatine kinase, AST: Aspartate aminotransferase, ALT: Alanine aminotransferase, ALP: Alkaline phosphatase, TT4: Total thyroxine.
(XLSX)

## References

[1] Hartmann K. Clinical aspects of feline immunodeficiency and feline leukemia virus infection. Vet Immunol Immunopathol. 2011;143(3):190–201. doi: 10.1016/j.vetimm.2011.06.003 21807418 PMC7132395

[2] Reinacher M. Diseases associated with spontaneous feline leukemia virus (FeLV) infection in cats. Vet Immunol Immunopathol. 1989;21(1):85–95. doi: 10.1016/0165-2427(89)90132-3 2549696 PMC7133624

[3] Little S, Levy J, Hartmann K, Hofmann-Lehmann R, Hosie M, Olah G, et al. 2020 AAFP Feline Retrovirus Testing and Management Guidelines. J Feline Med Surg. 2020;22(1):5–30. doi: 10.1177/1098612X19895940 31916872 PMC11135720

[4] Gleich S, Hartmann K. Hematology and Serum Biochemistry of Feline Immunodeficiency Virus-Infected and Feline Leukemia Virus-Infected Cats. J Vet Intern Med. 2009;23(3):552–8. doi: 10.1111/j.1939-1676.2009.0303.x 19645840

[5] Cotter SM, Hardy WD, Essex M. Association of feline leukemia virus with lymphosarcoma and other disorders in the cat. J Am Vet Med Assoc. 1975;166(5):449–54. 163223

[6] Stone AES, Brummet GO, Carozza EM, Kass PH, Petersen EP, Sykes J, et al. 2020 AAHA/AAFP Feline Vaccination Guidelines. J Feline Med Surg. 2020;22(9):813–30. doi: 10.1177/1098612X20941784 32845224 PMC11135662

[7] Louwerens M, London CA, Pedersen NC, Lyons LA. Feline Lymphoma in the Post—Feline Leukemia Virus Era. J Vet Intern Med. 2005;19(3):329–35. doi: 10.1892/0891-6640(2005)19[329:flitpl]2.0.co;2 15954547

[8] Osterhaus A, Weijer K, Uytdehaag F, Jarrett O, Sundquist B, Morein B. Induction of protective immune response in cats by vaccination with feline leukemia virus iscom. J Immunol. 1985;135(1):591–6. 2987351

[9] Lewis MG, Mathes LE, Olsen RG. Protection against feline leukemia by vaccination with a subunit vaccine. Infect Immun. 1981;34(3):888–94. doi: 10.1128/iai.34.3.888-894.1981 6277792 PMC350952

[10] Beall MJ, Buch J, Clark G, Estrada M, Rakitin A, Hamman NT, et al. Feline Leukemia Virus p27 Antigen Concentration and Proviral DNA Load Are Associated with Survival in Naturally Infected Cats. Viruses. 2021;13(2). doi: 10.3390/v13020302 33671961 PMC7919025

[11] Burling AN, Levy JK, Scott HM, Crandall MM, Tucker SJ, Wood EG, et al. Seroprevalences of feline leukemia virus and feline immunodeficiency virus infection in cats in the United States and Canada and risk factors for seropositivity. J Am Vet Med Assoc. 2017;251(2):187–94. doi: 10.2460/javma.251.2.187 28671491

[12] Little S, Sears W, Lachtara J, Bienzle D. Seroprevalence of feline leukemia virus and feline immunodeficiency virus infection among cats in Canada. Can Vet J. 2009;50(6):644–8. 19721785 PMC2684053

[13] O’Connor TP, Tonelli QJ, Scarlett JM. Report of the National FeLV/FIV Awareness Project. J Am Vet Med Assoc. 1991;199(10):1348–53. 1666080

[14] Westman ME, Malik R, Hall E, Norris JM. Diagnosing feline immunodeficiency virus (FIV) infection in FIV-vaccinated and FIV-unvaccinated cats using saliva. Comp Immunol Microbiol Infect Dis. 2016;46:66–72. doi: 10.1016/j.cimid.2016.03.006 27260813

[15] Shelton GH, Waltier RM, Connor SC, Grant C. Prevalence of feline immunodeficiency virus and feline leukemia virus infections in pet cats. J Am Anim Hosp Assoc. 1989;25:7–12.

[16] Levy JK, Scott HM, Lachtara JL, Crawford PC. Seroprevalence of feline leukemia virus and feline immunodeficiency virus infection among cats in North America and risk factors for seropositivity. J Am Vet Med Assoc. 2006;228(3):371–6. doi: 10.2460/javma.228.3.371 16448357

[17] IDEXX. Inc. SNAP* Combo FeLV/FIV Antibody Test (package insert). In: Maine: IDEXX Inc, editor. Maine: IDEXX Inc, 2017.

[18] Levy JK, Crawford PC, et al. American Association of Feline Practitioners Feline Retrovirus Management Guidelines. 2009. 10.1016/j.jfms.2008.03.002 PMC10832685 18455463

[19] Goldkamp CE, Levy JK, Edinboro CH, Lachtara JL. Seroprevalences of feline leukemia virus and feline immunodeficiency virus in cats with abscesses or bite wounds and rate of veterinarian compliance with current guidelines for retrovirus testing. J Am Vet Med Assoc. 2008;232(8):1152–8. doi: 10.2460/javma.232.8.1152 18412524

[20] Gleich SE, Krieger S, Hartmann K. Prevalence of feline immunodeficiency virus and feline leukaemia virus among client-owned cats and risk factors for infection in Germany. J Feline Med Surg. 2009;11(12):985–92. doi: 10.1016/j.jfms.2009.05.019 19616984 PMC11318771

[21] Arjona A, Escolar E, Soto I, Barquero N, Martin D, Gomez-Lucia E. Seroepidemiological Survey of Infection by Feline Leukemia Virus and Immunodeficiency Virus in Madrid and Correlation with Some Clinical Aspects. J Clin Microbiol. 2000;38(9):3448–9. doi: 10.1128/JCM.38.9.3448-3449.2000 10970400 PMC87403

[22] Cotter SM. Management of healthy feline leukemia virus-positive cats. J Am Vet Med Assoc. 1991;199(10):1470–3. 1666105

[23] Fabrizio F, Calam AE, Dobson JM, Middleton SA, Murphy S, Taylor SS, et al. Feline mediastinal lymphoma: a retrospective study of signalment, retroviral status, response to chemotherapy and prognostic indicators. J Feline Med Surg. 2014;16(8):637–44. doi: 10.1177/1098612X13516621 24366846 PMC11164164

[24] Weiser MG, Kociba GJ. Erythrocyte Macrocytosis in Feline Leukemia Virus Associated Anemia. Vet Pathol. 1983;20(6):687–97. doi: 10.1177/030098588302000604 6316617

[25] Werner LL, Turnwald GH, Willard MD. Immunologic and Plasma Protein Disorders. Small Animal Clinical Diagnosis by Laboratory Methods. 2004:290–305. doi: 10.1016/B0-72-168903-5/50017-3

[26] Scherk MA, Ford RB, Gaskell RM, Hartmann K, Hurley KF, Lappin MR, et al. 2013 AAFP Feline Vaccination Advisory Panel Report. J Feline Med Surg. 2013;15(9):785–808. doi: 10.1177/1098612X13500429 23966005 PMC11110975

